# Supplementary material for: LMNA R644C associates with hepatic steatosis in a large cohort and increases cellular lipid droplet accumulation in vitro
Source: medRxiv. 2023 Dec 22:2023.12.20.23300290. Preprint. [Version 1] doi: 10.1101/2023.12.20.23300290 (PMC10775342; doi:10.1101/2023.12.20.23300290)
Supplement: Supplement 1 [file media-1.pdf]

Table S1. Liver-related phenotypes associated with rs142000963-T in MGI.

Phenotype	Direction	*P*-value	Odds ratio [95% confidence interval]
Non-malignant ascites	Positive	0.002	5.0 [1.9-13.2]
Liver replaced by transplant	Positive	0.033	10.0 [1.2-86.1]
Acute and subacute necrosis of liver	Positive	0.046	16.4 [1.1-255.7]

Table S3. Association of rs142000963-T with extra-hepatic metabolic phenotypes (via the Type 2 Diabetes Knowledge Portal).

Phenotype	Direction	*P*-value
Waist to hip ratio	Positive	0.001
Type 2 diabetes	Positive	0.004
Hemoglobin A1c	Positive	0.001
HDL	Negative	0.004

Supplementary Methods

Cohort and Ethics Statement. Michigan Genomics Initiative (MGI) participants provided written informed consent approved by the University of Michigan IRB, which approved all human subjects research in this study.

Association analysis. We tested rs142000963 (GRCh38 g.156138719C>T; *LMNA* p.R644C) for effects on hepatic steatosis in the MGI cohort (freeze 3; N=57,022 individuals). Natural language processing of pathology and radiology reports was used to identify cases (n=5,856) with hepatic steatosis on liver biopsy or imaging; participants not classified as cases were considered controls (n=51,166). All included MGI participants had been genotyped via the Illumina HumanCoreExome array, which includes rs142000963; overall minor allele frequency was 0.002. Association analysis was performed in SAIGE v0.29 with steatosis as the outcome, controlling for age, age^2, sex, and the first 10 principal components in an additive genetic model.

Phenome-wide association study (PheWAS). PheWAS was performed for *LMNA* R644C (rs142000963-T) in MGI freeze 3 using a web-based browser (http://pheweb.org; last accessed 12/4/2023), with a focus on liver-related phenotypes. Liver-related phenotypes that significantly associated with rs142000963 (P<0.05) were included Supplementary Table 1. PheWAS of extra-hepatic metabolic traits was performed with publicly available data via the Type 2 Diabetes Knowledge Portal (T2DKP, https://t2d.hugeamp.org/ or type2diabetesgenetics.org, RRID SCR_003743; last accessed 12/11/2023)[1].

Cell culture and treatment conditions. Huh7 cells were maintained in DMEM containing glucose and lacking L-glutamine, supplemented with 10% heat-inactivated fetal bovine serum (Thermo Fisher A3840202, USA) at 37°C, 5% CO_2. For transfection, cells were plated at ~60-70% confluency one day prior to transfection. For lipid staining, cells were plated in 4-well

chamber slides in DMEM with 10% FBS, then transfected with mCherry-lamin A the next day. For lipid supplementation, culture medium was changed 24 hours post-transfection to DMEM containing 1% fatty acid–free bovine serum albumin (Sigma-Aldrich) and oleic acid (100 µM; Sigma-Aldrich), and cells were incubated for an additional 24 hours. For staining, cells were fixed in 4% paraformaldehyde (PFA) in PBS for 15 mins at 22°C, then washed in PBS; neutral lipids were stained with 10 µM boron dipyrromethene 493/503 (BODIPY; Thermo Fisher). Quantification of green BODIPY fluorescence was performed using ImageJ version 1.53t for at least three 20× fields per condition per experiment and expressed as total BODIPY fluorescence per field (CTCF) which was calculated as CTCF = Integrated Density – (Area of selected cells × Mean fluorescence of background readings).

Plasmids and transfection. Amino-terminally tagged human *LMNA* was described previously[2]. The *LMNA* open reading frame was cloned into mCherry-Parkin, Addgene plasmid #23956 (replacing the Parkin open reading frame), via the BspEI and BamHI sites. mCherry-Parkin was a gift from Richard Youle (Addgene plasmid #23956; http://n2t.net/addgene:23956; RRID:Addgene_23956)[3]. The R644C variant of *LMNA* was generated using the QuikChange II site-directed mutagenesis kit (Agilent Technologies, Santa Clara, CA). Lipofectamine-2000 (Life Technologies, Carlsbad, CA) was used for transfection of Huh7 cells according to the manufacturer's instructions.

Statistical analysis. For PheWAS testing, the Benjamini-Hochberg method was used to correct for simultaneous testing of multiple phenotypes, with false-discovery rate set to 0.05. Statistical analysis of *in vitro* data was performed in GraphPad Prism v.8. To compare >2 groups (Figure 1C), 1-way ANOVA was performed in Graphpad followed by Tukey's multiple comparison test.

Supplementary References

1. Costanzo MC, von Grotthuss M, Massung J, et al. The Type 2 Diabetes Knowledge Portal: An open access genetic resource dedicated to type 2 diabetes and related traits. Cell Metab 2023;35:695-710 e6.

2. Brady GF, Kwan R, Ulintz PJ, et al. Nuclear lamina genetic variants, including a truncated LAP2, in twins and siblings with nonalcoholic fatty liver disease. Hepatology 2018;67:1710-1725.

3. Narendra D, Tanaka A, Suen DF, et al. Parkin is recruited selectively to impaired mitochondria and promotes their autophagy. J Cell Biol 2008;183:795-803.
